# Interoceptive hunger, eating attitudes and beliefs

**DOI:** 10.3389/fpsyg.2023.1148413

**Published:** 2023-04-21

**Authors:** Richard J. Stevenson, Brayson J. Hill, Alannah Hughes, Madeline Wright, Johanna Bartlett, Supreet Saluja, Heather M. Francis

**Affiliations:** Department of Psychology, Macquarie University, Sydney, NSW, Australia

**Keywords:** hunger, interoception, individual difference, dysfunctional eating, beliefs

## Abstract

Interoceptive individual differences have garnered interest because of their relationship with mental health. One type of individual difference that has received little attention is variability in the sensation/s that are understood to mean a particular interoceptive state, something that may be especially relevant for hunger. We examined if interoceptive hunger is multidimensional and idiosyncratic, if it is reliable, and if it is linked to dysfunctional eating and beliefs about the causes of hunger. Participants completed a survey just before a main meal, with most retested around 1 month later. We found that interoceptive hunger has 11 dimensions, and while people differ considerably in their combinations of interoceptive hungers, these represent only 4% of all possible permutations. Hunger reports were reliable. We found relationships between variability in hunger interoception and dysfunctional eating, especially for uncontrolled eating. We also found that hunger beliefs were in some cases strongly related to aspects of hunger interoception. The implications of these findings are discussed.

## 1. Introduction

The perception of internal bodily events is termed interoception (Khalsa et al., 2018). Individual differences in interoception have been linked to many psychiatric conditions, especially eating disorders, depression, and anxiety (e.g., Harshaw, 2015; Khalsa et al., 2018). To date, interoceptive individual differences have focussed on four constructs (e.g., Garfinkel et al., 2015; Todd et al., 2021): (1) interoceptive accuracy (capacity to report an internal state that occurs); (2) interoceptive sensibility (confidence in reporting); (3) interoceptive awareness [the relationship between (1) and (2)] and (4) implicit measures of interoceptive processing—with (1–3) reflecting explicit processes. While important, these do not encompass all sources of interoceptive variation. One source that has received little attention, concerns variability in the interoceptive sensation/s that *constitute* a state. While this may have limited relevance for some states, it may be particularly pertinent for hunger (e.g., Stevenson et al., 2015). This is significant, as abnormalities in interoceptive hunger processing are implicated in all eating disorders, and this group of mental illnesses is not only economically costly—around 65 Billion US dollars in the United States alone (Streatfeild et al., 2021)—but is in urgent need of new treatment approaches.

Hunger has been conceptualized in two main ways (e.g., Cannon and Washburn, 1912). First, as an appetite for a specific palatable food, in response to an associated cue (e.g., seeing an advert for an ice-cream). Second, as a general desire to eat, arising either from an interoceptive (e.g., rumbling stomach) or temporal (e.g., “its lunch time”) cue. While appetite has been extensively studied, with several well-developed models (e.g., Lowe and Butryn, 2007; May et al., 2012; Papies et al., 2020), far less is known about the operation of temporal or interoceptive cues (Stevenson et al., 2023). For interoceptive cues, the focus here, there are clearly individual differences (see Stevenson et al., 2015). However, the nature and extent of these is poorly understood, partly because of a dearth of work and also from disagreements among the few studies to explore them.

Five studies have examined the interoceptive sensations that people report as hunger (Monello and Mayer, 1967; Garfinkel, 1974; Harris and Wardle, 1987; Friedman et al., 1999; Murray and Vickers, 2009). Two find hunger is multidimensional and idiosyncratic (Monello and Mayer, 1967; Harris and Wardle, 1987). Three find commonality, with abdominal-related sensations predominating (Garfinkel, 1974; Friedman et al., 1999; Murray and Vickers, 2009). These divergent findings leave three issues unresolved. First, are hunger sensations multidimensional? Second, if they are, *how* do people differ? Third, are such hunger reports reliable? To address these questions we recruited hungry participants and had them complete Monello and Mayer’s (1967) hunger sensations survey. Participants were then retested ~1-month later to assess reliability.

If hunger is reliably multidimension and idiosyncratic, two further questions arise. One concerns dysfunctional eating. As noted above, individual differences in interoception have been consistently linked to mental health, and particularly disordered eating (e.g., Harshaw, 2015; Khalsa et al., 2018). As a preliminary means of testing whether putative variability in hunger interoception is similarly important, we asked participants to complete the Three Factor Eating Questionnaire (TFEQ; Cappelleri et al., 2009). We tested if eating styles, which vary dimensionally from functional to dysfunctional, are associated with variation in interoceptive hunger.

Another question concerns the source of variability in interoceptive hunger. The body generates a range of internal sensations that can be interpreted in different ways (Pennebaker, 1982). This has been well-studied for pain and anxiety (e.g., Cioffi, 1991). People also hold beliefs about hunger, some of which are poorly supported by scientific data (e.g., Rogers and Brunstrom, 2016). Assanand et al. (1998) found that many believe that energy-depletion triggers interoceptive hunger, although there is in fact little support for this idea in the literature. This is because the body has very substantial energy stores and rarely in fact “runs low” on fuel (Rogers and Brunstrom, 2016). People who believe that energy-depletion causes hunger may readily heed interoceptive signals because they may be taken to indicate that they are “running low on fuel” (i.e., like a car and petrol). However, if one believed that hunger is triggered by environmental cues (i.e., learned), bodily signals may be less important. Beliefs about the causes of hunger might then explain some of the hypothesized variation in interoceptive hunger. To explore this, we developed a new hunger beliefs measure. This is an important issue to explore, as hunger is a central construct in the psychology of ingestive behavior, and is regarded by lay people as a key trigger for eating, yet little is known about beliefs regarding the causes of hunger nor about its putative links to interoceptive hunger.

In sum, the study aimed to determine if hunger sensations are multidimensional, and if they are, how people differ in this regard, and whether such differences are reliable. No study has explored these types of individual difference in interoceptive hunger before, nor determined their reliability. To initially assess their potential relevance to eating disorders, we also examined whether these individual differences in interoceptive hunger are related to attitudinal dimension that merge into disordered eating, as well as with beliefs about the causes of hunger. Both issues have seen little prior exploration. Practically, individual differences in hunger cannot only serve as new potential markers for disordered eating, they may also point to new treatment modalities, as we examine in the discussion. Theoretically, hunger is a central construct in biological psychology (e.g., Rogers and Brunstrom, 2016; Stevenson et al., 2023), yet many of its basic features remain unexplored, most notably the nature of interoceptive hunger and its variability, which are explored here.

## 2. Method

### 2.1. Participants

A sample size of ~200 was the aim, based upon the observation that prior studies observing multidimensionality were those with larger sample sizes (i.e., >100 participants; Monello and Mayer, 1967; Harris and Wardle, 1987). In contrast, the smaller studies (i.e., <100 participants; Garfinkel, 1974; Friedman et al., 1999; Murray and Vickers, 2009) tended toward uni-dimensionality. In addition, 200 participants represents an adequate sample size for factor analysis (e.g., Tabachnick and Fidell, 1989).

Potential participants were asked to take part only if they had no history/current eating disorder and no medical condition affecting hunger. 191 university students started the survey, with 185 completing part 1, and 108 completing part 1 and 2. Five were excluded due to failing all four check questions (see below) or reporting an eating disorder, leaving 180 for the analysis of part 1 (147 female; M BMI = 23.5 [SD = 5.4]; M age = 21.5 [SD = 7.0]), and 107 for part 1 and 2 (90 female; M BMI = 23.8 [SD = 6.3]; M age = 22.2 [SD = 7/6]). The study was approved by the Macquarie University IRB (Project ID 11337) and consent was provided by each participant.

### 2.2. Materials

#### 2.2.1. Hunger survey

The survey used Monello and Mayer’s (1967) hunger questions (i.e., items) but excluded those concerning satiety. The final survey was composed of 48 items, grouped into seven blocks. The first dealt with current hunger and asked about time since the last meal, current hunger, urge to eat, preoccupation with food, and how much one could eat now. Responses were made on five-point category scales (no response to extremely/strong) except for the time question, which had 4 time-intervals since last eating.

The six principal blocks followed, each using a similar form “When you are hungry, which of the following sensations do you experience?”. These sensations were grouped into stomach, with 9 items; mood, 9; mouth, 7; throat, 7; head, 3; and general, 8 (see Monello and Mayer, 1967, for details). While Monello and Mayer (1967) used just two response categories (present/absent), we asked participants to make their judgment on a six-point category scale, with 1 = not at all, 2 = very weak, 3 = weak, 4 = moderate, 5 = strong and 6 = very strong. This approach was adopted to discern if perhaps only a few interoceptive cues would emerge if participants could indicate their intensity.

#### 2.2.2. Beliefs survey

Participants were asked to judge their agreement/disagreement [1 (strongly disagree) to 4 (neither agree or disagree) to 7 (strongly agree)] with 43 statements. These were divided into 23 concerning homeostatic views of hunger (e.g., I believe that when I am hungry I have low levels of blood sugar; a craving for a certain food means that I am missing certain nutrients from my body) and 20 concerning learning/environmental influences on hunger (e.g., the sight of food I like activates my hunger; I believe that sitting in a restaurant would increase my hunger). Items with poor reliability were deleted leaving 20 homeostatic items with a Cronbach’s alpha = 0.76 and 15 learning/environmental items with an alpha = 0.64. These fall in the acceptable range for research-related instruments (i.e., 0.5+, Nunnally, 1978). Each respective set of items was averaged, generating a homeostatic score and a learning/environment score. These scores did not significantly correlate (*r* = 0.14). Intraclass correlation coefficients were then examined for the homeostatic and learning/environment scores between the first and second administration of this survey. The ICCs were, homeostatic = 0.70 and learning/environment = 0.62, indicating “good” to “fair-to-good” reliability (Fleiss, 1986; Cicchetti, 1994).

#### 2.2.3. TFEQ

The revised 18 item TFEQ (Cappelleri et al., 2009) was used, having good internal reliability (*α* = 0.78–0.94).

#### 2.2.4. Check questions

Four check questions were included to determine participant attention. Two were embedded in the hunger survey (loss of vision as a hunger sign; people need food to survive) and two in the beliefs survey (breakfast is normally eaten in the evening; sugar has a sweet taste).

### 2.3. Procedure

In part 1, participants were instructed to complete the survey while hungry 30 min before their main meal (as with part 2). The survey took 20–25 min to complete. Following consent, and biographic information (name, age, height, weight, gender, currently dieting, any condition that might affect hunger), the hunger survey, the beliefs survey and the TFEQ were completed.

Part 2 was undertaken a mean of 27.4 days later (SD = 7.4, range = 18–56 days). Participants repeated their biographic details (to enable matching), and the hunger and beliefs surveys.

These data are available in Supplementary material.

### 2.4. Analysis

Data were suitable for parametric analysis, after reviewing skewness and kurtosis, and examining for outlying observations. Alpha was set at 0.05 with Bonferroni adjustment for multiple comparisons.

## 3. Results

### 3.1. Current hunger

Participants reported being hungry in both part 1 and 2 of the study. For part 1, median time since last eating was 2–4 h, with moderate hunger (*M* = 2.8/5, SD = 0.9), moderate urge to eat (*M* = 2.9/5, SD = 0.9), small to moderate thoughts about food (*M* = 2.6/5, SD = 0.9) and desire for a moderate serving size now (*M* = 3.0, SD = 0.8). For part 2, median time since last eating was 2–4 h, with moderate hunger (*M* = 2.8/5, SD = 0.9), moderate urge to eat (*M* = 2.8/5, SD = 0.8), small to moderate thoughts about food (*M* = 2.5/5, SD = 0.9) and desire for a moderate serving size now (*M* = 3.0, SD = 0.7).

### 3.2. Is hunger multidimensional?

The 403 items from the principal part of the hunger survey were factorable, as KMO index exceeded 0.6 (KMO = 0.86) and Bartlett’s test was significant (chi-squared[903] = 4,157.9, *p* < 0.001). Eleven factors with an eigen value >1 were extracted following promax rotation (see Supplementary material, Supplementary Table S1 for structure matrix). Out of the 11 factors (cumulative variance = 67.9%), four were based around specific bodily locations, namely factor 1 oropharynx (26.0% variance), factor 8 stomach fullness (2.7% variance), factor 9 cold empty stomach (2.5% variance), and factor 11 salivation (2.3% variance). One was a diffuse bodily location, factor 4 (4.4% variance), capturing fatigue. Six were characterized by their affective tone. Factors 2 (12.4% variance), 6 (3.2% variance), 7 (3.1% variance) and 10 (2.5% variance) were affectively negative. Factors 3 (5.1% variance) and 5 (3.7% variance) were positive. These findings suggest that interoceptive hunger is multidimensional.

We then constructed mean scores (Table 1) for each factor (if required) and compared them using a one-way repeated measures ANOVA. The ANOVA revealed a significant main effect of factor (*F*(10,1790) = 171.83, MSE = 0.94, *p* < 0.001, partial eta-squared = 0.50). Participants judged the strongest interoceptive hunger signal to be cold empty, which was judged more intense than fatigue and irritable, which in turn were more intense than salivation, bored and nausea. So, while hunger is multidimensional, the dominant interoceptive sensation—cold empty—is jointly characterized by an absence of bodily warmth and an empty stomach.

**Table 1:** Mean scores for each of the factors and their rating equivalent.

Factor name (number of items)	Mean intensity score (SD)	Label equivalent to the intensity score	Bonferroni adjusted contrasts^a^
1. Oropharynx (7)	3.2 (1.2)	Weak	2–5, 7–11
2. Nausea (8)	3.5 (1.1)	Weak/moderate	1, 3–9
3. Positive feeling (6)	2.1 (0.8)	Very weak	1–2, 4–7, 9–11
4. Fatigue (6)	4.3 (1.0)	Moderate	1–3, 5–11
5. Positive mood (4)	2.5 (0.9)	Very weak/weak	1–4, 6–11
6. Cold tension (3)	3.0 (1.2)	Weak	2–5, 7–11
7. Irritable (3)	4.0 (0.9)	Moderate	1–6, 8–10
8. Stomach fullness (2)	1.9 (1.0)	Very weak	1–2, 4–7, 9–11
9. Cold empty (2)	5.0 (0.7)	Strong	1–8, 10–11
10. Bored (1)	3.6 (1.5)	Weak/moderate	1, 3–9
11. Salivation (1)	3.8 (1.3)	Weak/moderate	1, 3–6, 8–9

a Listed factor numbers significantly differ from that factor.

### 3.3. How do people differ in interoceptive hunger?

For each participant we counted how many hunger factors exceeded a mean intensity score of 4 (moderate), which we assumed meant the interoceptive hunger factor would be relatively distinct (see Table 2). We note that using a higher [i.e., 5 (strong)] or lower [i.e., 3 (weak) or 2 (very weak)] intensity score produces substantially similar outcomes (and resultant correlations), suggesting the stability of this approach.

**Table 2:** Individual differences in interoceptive hunger.

Number of factors exceeding moderate intensity	Number of participants (% of total)	Number of different combinations of factors (number possible)	Commonest combination of factors (and commonality as observed % of total for *just* that row)
0	2 (1.1)	N/A	N/A
1	5 (2.8)	1 (11)	F9 (100%)
2	22 (12.2)	8 (55)	F9, 11 (55%)
3	34 (18.9)	14 (165)	F9, 10, 11 (24%)
4	33 (18.4)	13 (330)	F4, 7, 9, 11 (27%)
5	30 (16.7)	19 (462)	F4, 7, 9, 10, 11 (23%)
6	23 (12.8)	14 (462)	F2, 4, 7, 9, 10, 11 (17%)
7	22 (12.2)	9 (330)	F1, 2, 4, 7, 9, 10, 11 (27%)
8	6 (3.4)	1 (165)	F1–7, 9, 10, 11 (100%)
9	2 (1.1)	2 (55)	F1–8, 10 (50%) & F2, 4–11 (50%)
10	1 (0.6)	1 (11)	F1, 2, 4–11 (100%)
11	0 (0.0)	N/A (1)	N/A

We then computed the number of combinations of hunger factors, starting with those who had two factors exceeding moderate intensity, then those with three, and so on (see Table 2). While there were many different combinations of hunger factors exceeding moderate intensity (82 identified), theoretically, 2,047 are possible, and so 82 is only 4% of this total. This combinatorial constraint reflects some agreement between participants as to which hunger factors are important. The commonest combinations tended to include a focal bodily cue (F9, F11), a diffuse bodily cue (F4), and negative affective states (F7, F10). These findings suggest that people do differ both in terms of the number of interceptive hunger signals they identify, and in their combination.

### 3.4. Stability

The stability of the hunger survey was tested on a participant-by-participant basis, by correlating each participant’s 43 responses from part 1, with their corresponding responses from part 2. The mean correlation (Pearson’s *r*) across the 107 participants who completed repeat testing was = 0.66 (SD = 0.16). Normalized values of *r* (*r*′) were compared to a mu of zero using a one-sample *t*-test, to determine if participants had, overall, positive correlations between their two responses. This was confirmed (*t*(106) = 30.50, *p* < 0.001, *r*^2^ = 0.90).

The ICCs for individual factors were: oropharynx = 0.68, nausea = 0.75, positive feeling = 0.42, fatigue = 0.69, positive mood = 0.52, cold tension = 0.69, irritable = 0.57, stomach fullness = 0.55, cold empty = 0.57, bored = 0.50 and salivation = 0.31. Except for salivation, these values indicate “good” to “fair-to-good” reliability (Fleiss, 1986; Cicchetti, 1994).

### 3.5. Relationship to eating attitudes (TFEQ) and beliefs

Each TFEQ scale was correlated with one derived measure reflecting interoceptive hunger diversity, namely the number of factors exceeding moderate intensity (number of hungers), with each of the hunger factors, and with the highest hunger intensity score irrespective of factor (maximal hunger; see Table 3). Greater Uncontrolled eating was associated with having more forms of interoceptive hunger, and with two interoceptive hunger factors, fatigue and bored. Greater restraint was linked to cold empty, and greater emotional eating with irritable.

**Table 3:** Correlations (Pearson) between interoceptive hunger, eating attitudes and beliefs.

Variable	Eating attitudes (TFEQ)			Beliefs about hunger	
	Restraint eating	Uncontrolled eating	Emotional	Homeostatic	Learning/environment
Number of hungers^b^	0.11	0.22^a^	0.10	0.29^a^	0.26^a^
Maximal hunger	0.09	0.14	0.10	0.08	0.11
F1. Oropharynx	0.13	0.18	0.04	0.31^a^	0.18
F2. Nausea	0.11	0.13	0.09	0.29^a^	0.25^a^
F3. Positive feeling	−0.02	−0.02	−0.07	0.01	0.05
F4. Fatigue	0.17	0.20^a^	0.11	0.44^a^	0.16
F5. Positive mood	−0.04	−0.09	−0.12	−0.01	−0.06
F6. Cold tension	0.14	0.16	0.05	0.23^a^	0.20^a^
F7. Irritable	0.09	0.13	0.20^a^	0.33^a^	0.15
F8. Stomach fullness	0.11	0.04	−0.01	0.00	0.03
F9. Cold empty	0.19^a^	0.05	0.09	0.03	−0.01
F10. Bored	0.07	0.21^a^	0.17	0.04	0.29^a^
F11. Salivation	−0.04	0.07	−0.03	0.04	0.13

a *p* < 0.01 (Bonferroni corrected by row [0.05/5]).

b These relationships remain significant if maximal hunger is partialled out.

### 3.6. Beliefs about hunger

Both homeostatic (*M* = 4.9, SD = 0.9) and learning/environment (*M* = 4.7, SD = 0.6) mean scores significantly exceeded 4 (i.e., neither agree nor disagree; *t*’s > 15.24), indicating agreement with both these perspectives on hunger. However, homeostatic items were agreed with to a greater extent than learning-related perspectives (*t*(179) = 3.43, *p* < 0.001).

Correlations with belief scores are presented in Table 3. Greater number of hungers was linked to more strongly held homeostatic and learning/environment beliefs. Greater homeostatic beliefs were associated with 5 factors and greater learning/environment beliefs with 3—with 2 of these overlapping—cold tension and nausea.

## 4. Discussion

People differ in how many interoceptive hunger cues are relevant to them (as indicated by their intensity) and in the combinations they possess. However, the range of combinations is constrained, representing only 4% of all permutations, with each tending to include a focal, a diffuse, and a negatively affective toned interoceptive hunger. Interoceptive hunger reports were generally stable, both when examined on a participant-by-participant basis and from ICCs of each factor. There was evidence for relationships between interoceptive hunger and TFEQ dimensions, particularly uncontrolled eating. Both homeostatic and learning/environmental beliefs about the causes of hunger were related to various facets of interoceptive hunger.

One potential limitation concerns the predominance of female participants. Monello and Mayer (1967) found that interoceptive hunger tended to be more intense for females. In contrast, Harris and Wardle (1987) found no gender differences. While this topic deserves closer scrutiny, especially as some forms of eating dysfunction are linked to gender, our main conclusions are probably not affected by our sample.

A further limitation concerns our manipulation of hunger, with participants asked to complete the survey 30 min before a main meal. While participants did report being hungry at the time the survey was completed, there was no way to confirm adherence to the protocol other than by this self-report. How hungry a participant is when they complete the survey might potentially alter the outcome. However, prior findings do not strongly suggest this. Monello and Mayer (1967) did not observe any major differences when participants were asked to complete their survey mildly, moderately or strongly hungry—but critically, these states were imagined. Harris and Wardle (1987) did manipulate hunger and obtained similar results to Monello and Mayer (1967). So, while degree of hunger may be important, its variation is probably not critical to the conclusions of this study.

The beliefs survey was designed to assess the degree to which people possess homeostatic and learning-related beliefs about the causes of hunger. As only one prior study has explored this (Assanand et al., 1998), there was no validated measure on which we could draw. Consequently, we designed a preliminary questionnaire to address participant beliefs. As we discuss further below, this was found to be related to the nature of participants interoceptive hunger. However, we note that this scale remains to be validated against other survey measures (e.g., power of food scale; Lowe et al., 2009) and behavior (e.g., response to actual food deprivation). Notwithstanding this, we did obtain preliminary data that this measure is reliable over an ~1-month re-test interval and that it has adequate internal reliability.

A key study finding was that interoceptive hunger cues are multidimensional, consistent with the earlier reports of Monello and Mayer (1967) and Harris and Wardle (1987). An additional observation was that this multidimensional pattern was reliable over a 1-month re-test interval, suggesting that it is not the result of variable or noisy responding. The data also revealed that participants hunger cues are quite idiosyncratic, in that people differed in the number of these interoceptive cues they reported. This seems to be quite different to other interoceptive systems, such as thirst, micturition, or defecation related sensations, which appear to be far less variable (e.g., Brunstrom et al., 2000; Halani et al., 2020; Langfield et al., 2022).

The idiosyncratic nature of interoceptive hunger cues suggests the possibility that some of this variation might arise from childhood learning (Bruch, 1969; Stevenson et al., 2023). For example, if a child’s stomach rumbled, a caregiver might remark “you must be hungry”. If the child then ate, and found that food tasted good, this would strengthen the association. There does not seem to be any data that bears upon this, perhaps because it is assumed internal states are “hardwired”. However, as several theorists have indicated, this assumption is probably incorrect, as the meaning of interoceptive sensations, even ones central to survival like feeding, are probably learned (e.g., Bruch, 1969; Harshaw, 2008). An additional source of variation here may be genetic (e.g., Herle et al., 2021; Warkentin et al., 2022), as observed in several prior studies, and as suggested before as a possible explanation for individual differences in hunger (Stevenson et al., 2015).

One reason why interoception has been of interest, is because of its links to psychiatric illness (e.g., Harshaw, 2015; Khalsa et al., 2018). Consistent with this we found that uncontrolled eating was significantly associated with three variables; more types of hunger, fatigue, and boredom. Uncontrolled eating is linked to binging (e.g., Bryant et al., 2019), and more hungers could provide more binge cues. Restraint was found to be associated with cold empty, perhaps reflecting past episodes of food-deprivation, and irritability was linked to emotional eating. There is a great deal of interest in trying to improve peoples’ capacity to experience hunger (and satiety), and especially in those living with obesity (e.g., Boutelle et al., 2020). This would make studying variability in interoceptive hunger an important exercise in this group, in addition to deciding which type of interoceptive hunger cue might be the easiest to learn and the most useful to possess.

Lay theories of psychological function have been found to be important determinants of behavior in several domains (Zedelius et al., 2017), but have received little attention in the context of hunger and appetite (Assanand et al., 1998). Hunger beliefs were associated with several facets of interoceptive hunger. We expected that homeostatic beliefs would be correlated with bodily states *believed* to indicate low fuel, and the correlation with fatigue (and irritability) is consistent with this. Some learning related beliefs might reflect knowledge that certain states come to be linked with hunger—boredom being one example. These findings indicate that interoceptive hunger relates to theories that people hold, even when these theories do not accord with what is currently known. This may have broader implications for appetite, which warrant exploration, particularly in regard to dieting and responsiveness to interoceptive cues.

As was noted in the Introduction, eating disorders have significant economic (e.g., Streatfeild et al., 2021) and personal impacts (e.g., Gilsbach and Herpertz-Dahlmann, 2023). One approach to treatment, for people who overeat, is to teach them to experience hunger and to use this to guide as to when to eat. The current research has two implications for this approach. The first is conceptual, in that the interoceptive cues that people are using are not reflections of a biological need to eat (i.e., homeostatic energy depletion models)—even though many of our participants believed this to be the case—but instead simply signal that food will taste good (be rewarding) now (e.g., Davidson and Stevenson, 2022). The second, and as exemplified by the multidimensional nature of interoceptive hunger, is which cue would a participant be best advised to focus on and how? Part of the problem is that people living with obesity, who often overeat, may have somewhat poorer interoceptive capacity than lean individuals (Robinson et al., 2021). Consequently, it might be easier to try and train them to use external temporal cues (i.e., clock time) as signals to eat (or not). With training, temporal cues may be as effective as interoceptive cues, as both could come to serve as feature positive occasion setters (Fraser and Holland, 2019)—that is cues that indicate food will be good to eat now. The effectiveness of such temporal training remains to be explored.

In conclusion, we found that interoceptive hunger cues are multidimensional, that people differ in how many of these cues they have, and that these differences are reliable. This is the first study to identify reliable individual differences in the cues that constitute interoceptive hunger. Importantly, and in addition, this study is the first to examine the potential relevance of these individual differences to eating disorders, finding a significant association with uncontrolled eating. The study also provides the first evidence of associations between participant beliefs about the causes of hunger and their experience of interoceptive hunger. Our results have several implications, both theoretical, for understanding how interoceptive hunger develops, and the possible role of learning in this process, and practically, for addressing the significant economic and social problem of disordered eating.

## Data availability statement

The original contributions presented in the study are included in the article/Supplementary material, further inquiries can be directed to the corresponding author.

## Ethics statement

The studies involving human participants were reviewed and approved by Macquarie University Human Research Ethics Committee. The patients/participants provided their written informed consent to participate in this study.

## Author contributions

All authors listed have made a substantial, direct, and intellectual contribution to the work and approved it for publication.

## Conflict of interest

The authors declare that the research was conducted in the absence of any commercial or financial relationships that could be construed as a potential conflict of interest.

## Publisher’s note

All claims expressed in this article are solely those of the authors and do not necessarily represent those of their affiliated organizations, or those of the publisher, the editors and the reviewers. Any product that may be evaluated in this article, or claim that may be made by its manufacturer, is not guaranteed or endorsed by the publisher.
