# Supplementary material for: Interoceptive hunger, eating attitudes and beliefs
Source: Front Psychol. 2023 Apr 21;14:1148413. doi: 10.3389/fpsyg.2023.1148413 (PMC10160651; doi:10.3389/fpsyg.2023.1148413)
Supplement: Supplementary file 1 [file Table_1.DOCX]

Table S1: Structure matrix for the hunger survey data

Factor Factor-item correlations for each factor

High loading items 1 2 3 4 5 6 7 8 9 10 11

**Factor 1 - Oropharynx**

Throat – Unpleasant feeling **0.86** 0.44 0.16 0.52 -0.06 0.29 0.19 0.02 -0.01 0.37 -0.08

Throat – Emptiness **0.80** 0.29 0.23 0.55 0.00 0.21 0.15 0.03 0.16 0.26 0.07

Throat – Tightness **0.78** 0.30 0.21 0.36 0.07 0.46 0.17 0.12 -0.13 0.20 0.04

Throat – Dry **0.72** 0.38 0.25 0.46 0.03 0.21 0.30 0.07 0.03 0.19 -0.08

Mouth – Emptiness **0.71** 0.33 0.45 0.57 -0.10 0.28 0.12 -0.24 0.10 0.23 -0.23

Mouth – Tightness **0.66** 0.42 0.48 0.41 0.03 0.51 0.20 -0.01 -0.11 0.07 -0.03

Mouth – Dry **0.63** 0.41 0.33 0.33 -0.08 0.18 0.31 -0.24 0.09 0.35 -0.37

**Factor 2 – Nausea**

Head – Dizzy 0.30 **0.81** 0.05 0.49 -0.14 0.28 0.30 0.06 0.04 0.22 -0.04

Stomach – Nausea 0.36 **0.79** 0.03 0.32 -0.05 0.37 0.25 0.09 0.01 0.22 0.10

Head – Headache 0.37 **0.77** 0.04 0.40 -0.05 0.17 0.15 0.19 0.25 0.50 -0.16

Throat – Nausea 0.62 **0.72** 0.27 0.50 0.03 0.40 0.33 0.15 -0.13 0.28 0.01

Stomach – Ache 0.48 **0.70** 0.24 0.36 0.16 0.54 0.08 0.02 0.10 0.34 0.01

Head – Faint 0.28 **0.64** 0.08 0.51 -0.04 0.11 0.31 -0.05 -0.17 0.19 0.01

Mouth – Unpleasant taste 0.54 **0.55** 0.33 0.44 -0.12 0.25 0.22 -0.14 -0.08 0.40 0.01

Stomach – Rumbling 0.39 **0.52** 0.15 0.36 0.11 -0.08 0.25 -0.32 0.36 0.26 -0.10

**Factor 3 – Positive feeling**

General – Energetic 0.21 -0.07 **0.79** 0.05 0.38 0.31 0.13 0.08 -0.28 -0.06 -0.04

Mouth – Relaxed 0.14 -0.03 **0.73** 0.05 0.38 0.10 -0.16 0.28 -0.04 0.14 0.06

Throat – Relaxed 0.08 -0.08 **0.72** 0.03 0.36 0.01 -0.09 0.11 -0.20 -0.04 -0.06

Stomach – Relaxed 0.09 0.03 **0.65** -0.08 0.42 0.02 -0.21 0.33 0.01 0.11 0.04

Throat – Pleasant feeling 0.45 0.11 **0.54** 0.36 0.35 0.22 -0.24 0.41 -0.39 0.10 0.01

Mouth – Pleasant taste 0.37 0.14 **0.49** 0.27 0.33 0.30 -0.03 0.47 -0.21 0.22 0.04

**Factor 4 - Fatigue**

General – Fatigued 0.54 0.46 0.08 **0.82** -0.24 0.20 0.17 0.11 0.22 0.36 -0.09

General – Weak 0.48 0.61 0.16 **0.80** -0.11 0.21 0.36 0.06 0.07 0.23 0.02

General – Sleepy 0.49 0.49 0.08 **0.75** -0.28 0.13 0.20 0.24 0.28 0.49 -0.04

General – Inattentive 0.43 0.37 0.15 **0.75** -0.07 0.29 0.44 -0.19 -0.07 0.13 -0.12

General – Restless 0.42 0.38 0.17 **0.72** 0.04 0.35 0.34 -0.09 0.06 0.22 0.02

Stomach – Hollow 0.47 0.37 0.13 **0.51** -0.03 0.39 0.22 -0.15 0.48 0.14 -0.24

**Factor 5 – Positive mood**

Mood – Cheerful 0.02 -0.10 0.34 -0.09 **0.86** 0.05 -0.02 0.12 -0.13 -0.05 0.05

Mood – Relaxed 0.08 0.03 0.44 -0.04 **0.78** 0.04 -0.29 0.26 0.05 0.11 -0.01

Mood – Content 0.14 0.12 0.37 -0.07 **0.74** -0.18 -0.20 0.39 0.01 0.27 -0.13

Mood – Excited -0.04 -0.05 0.32 -0.05 **0.69** 0.20 0.15 -0.06 -0.22 -0.09 0.21

**Factor 6 – Cold tension**

Stomach – Tension 0.46 0.46 0.19 0.41 0.12 **0.71** 0.03 0.05 0.18 0.33 -0.08

Mood – Nervous 0.33 0.44 0.19 0.35 -0.10 **0.68** 0.43 0.01 -0.10 0.14 0.09

General – Cold 0.55 0.31 0.24 0.41 -0.13 **0.59** 0.26 0.06 -0.19 0.33 -0.16

**Factor 7 – Irritable**

Mood – Impatient 0.25 0.27 -0.05 0.35 -0.14 0.10 **0.76** -0.13 0.06 0.08 -0.06

Mood – Irritable 0.23 0.48 -0.02 0.38 -0.14 0.29 **0.61** 0.10 -0.02 0.45 0.22

Mood – Apprehensive 0.27 0.30 0.31 0.50 0.26 0.43 **0.53** -0.03 0.01 0.05 -0.10

**Factor 8 – Stomach fullness**

Stomach – Full 0.11 0.13 0.40 0.02 0.26 -0.05 -0.16 **0.67** -0.16 0.13 -0.05

Stomach – Bloated 0.32 0.32 0.31 0.30 0.16 0.30 0.25 **0.54** -0.07 0.12 -0.01

**Factor 9 – Cold empty**

General – Warm 0.51 0.32 0.48 0.41 0.18 0.34 0.31 0.06 **-0.57** 0.22 -0.07

Stomach – Empty 0.33 0.23 0.22 0.47 -0.06 0.17 0.47 -0.18 **0.53** 0.21 -0.01

**Factor 10 – Bored**

Mood – Bored 0.31 0.30 0.19 0.26 0.10 0.21 0.13 0.03 0.02 **0.83** -0.02

**Factor 11 – Salivation**

Mouth – Salivation 0.11 0.10 0.10 0.11 0.12 0.09 0.06 0.02 -0.05 0.06 **0.88**
